# Recurrent pelvic organ prolapse after hysterectomy; a 10-year national follow-up study

**DOI:** 10.1007/s00404-024-07615-x

**Published:** 2024-07-08

**Authors:** Tea Kuittinen, Maarit Mentula, Sari Tulokas, Tea Brummer, Jyrki Jalkanen, Eija Tomas, Juha Mäkinen, Jari Sjöberg, Päivi Härkki, Päivi Rahkola-Soisalo

**Affiliations:** 1 Women’s Clinic, University of Helsinki and Helsinki University Hospital, Haartmaninkatu 2, 00290 Helsinki, Finland; 2 Central Hospital Østfold, Grålum, Norway; 3 Hospital Nova of Central Finland, Jyväskylä, Finland; 4 University of Turku, Turku, Finland; 5 University of Tampere and Tampere University Hospital, Tampere, Finland

**Keywords:** Pelvic organ prolapse, Laparoscopy, Hysterectomy, Surgical Techniques, Urogynecology

## Abstract

**Purpose:**

Hysterectomy may be a risk factor for pelvic organ prolapse (POP). We assessed the risk of recurrent POP (operations and visits) after hysterectomy among women with previous POP. We also studied patient and operation related risk factors for POP recurrence.

**Methods:**

This retrospective cohort study included 1697 women having previous POP diagnosis or POP at the time of hysterectomy (FINHYST 2006 cohort). Follow-up was until the end of 2016. The data was derived from the Finnish National Care register linked to the cohort. Hysterectomy approaches and other demographics were compared to the risk of a prolapse diagnosis and/or surgery. Cox regression model was used to identify hazard ratios.

**Results:**

Following hysterectomy, a total of 280 women (16.5%) had a POP reoperation and 359 (21.2%) had an outpatient visit due to POP. Vaginal vault prolapse repair was the most common POP reoperation (n = 181, 10.7%), followed by anterior wall repair (n = 120, 7.1%). Median time to POP reoperation was 3.7 years. Hysterectomy approach did not affect reoperations or visits. Previous cesarean section and anterior repair during hysterectomy were associated with decreased risk, whereas concomitant sacrospinous fixation and uterus prolapse as the main indication led to increased risk of anterior/vault prolapse reoperations. Concomitant posterior repair decreased posterior reoperations and visits, but uterus weight over 500 g caused a fivefold increased risk of posterior prolapse visit. Residential status was associated with elevated risk of any POP reoperations and visits.

**Conclusions:**

Approximately one out of five women suffering from POP ensue POP reoperation or visit after hysterectomy. These high rates are independent on hysterectomy approach, but probably indicate that hysterectomy may worsen previous pelvic floor dysfunction.

## What does this study add to the clinical work

**Table:** 

Approximately 20% of 1697 women who underwent hysterectomy for POP, experienced a subsequent POP reoperation or outpatient visits. High rates, irrespective of hysterectomy approach, suggest that hysterectomy may exacerbate known pelvic floor dysfunction. Additionally, apical repair was the primary reoperation (10.7% overall, 8,8% within the same compartment) highlighting the need for vaginal vault suspensions during hysterectomy.	

## Introduction

Pelvic organ prolapse (POP) ensue when supporting structures, pelvic floor muscles, fascia and ligaments are weakened by different risk factors such as pregnancies, deliveries, multiparity, congenital connective tissue abnormalities, ageing, menopause and obesity [1–3]. Approximately 1 out of 4 US women has some pelvic floor disorder with a 12.6% lifetime likelihood for POP surgery [4]. With a similar lifetime risk of undergoing POP surgery in Finland (13%), the risk for POP reoperation during a ten-year follow-up is 10.8% [5]. However, it is estimated that the prevalence of POP will rise by 46% over the next decades due to the ageing in population [6].

Hysterectomy is one of the most frequently performed gynecological procedure (30% by the age of 60 in the USA) for benign indications such as abnormal uterine bleeding, POP, fibroids, and endometriosis [7]. The impact of hysterectomy on subsequent POP is under debate. Previous studies have suggested that hysterectomy per se elevates the risk of developing POP later in life [8–11], particularly when prolapse was the primary indication for the procedure [8, 10, 12]. Conversely, conflicting findings have been reported in large epidemiological studies [13, 14] indicating that concurrent POP repair at the time of index hysterectomy protects against subsequent POP. Furthermore, the effect of hysterectomy approach on POP recurrence remains uncertain.

Most previous studies regarding the risk of POP recurrence have limitations. These studies did not assess outpatient visits [8, 10, 12–15], failed to evaluate different hysterectomy approaches [8, 12, 15], exclusively focused on primary prolapses [9, 11], lacked information on key variables such as parity, vaginal deliveries, body mass index (BMI) or prior POP surgery [10, 13–15] and lacked long-term follow-up exceeding 10 years [13, 14]. These important factors can significantly influence the risk of POP recurrence following hysterectomy.

Therefore, our primary objective in this retrospective cohort study with long-term follow-up was to ascertain the frequencies of reoperations and outpatient clinical visits related to POP in women who had undergone hysterectomy for POP. We also examined the association of subsequent POP with the hysterectomy approach and other surgical and patient-related risk factors.

## Methods

Our current study assessing the recurrence of POP following POP associated hysterectomy, represents a sub-analysis of the extensive nationwide prospective FINHYST cohort. This cohort involved 5279 women who underwent hysterectomy for benign indications. This study was conducted across 53 Finnish hospitals in 2006 and encompassed approximately 79% of all benign hysterectomies performed in Finland during that year. The study was included in the ClinicalTrials.gov protocol, and written consent was obtained from each participating patient. Throughout the study period gynecological surgeons completed paper questionnaires on patient characteristics and surgical data (Brummer et al. [16]). The primary indications for hysterectomy were determined by these gynecological surgeons, as outlined in Table 1. Patient and operation characteristics were defined as previously described [16].

**Table 1:** Demographics of the 1697 women undergoing hysterectomy 2006 and included in this study

Type of hysterectomy	All hyst^a^	AH^b^	LH	LAVH	VH
	1697 (100)	46 (2.7)	70 (4.1)	47 (2.8)	1534 (90.4)
Pre-operative patient factors					
Age, mean (sd)	59.2 (11.6)	57.8 (13.0)	55.6 (11.6)	56.8	59.4 (11.6)
Age, *n* (%)					
Under 45	185 (10.9)	6 (13.0)	8 (11.4)	8 (17.0)	163 (10.6)
45–54	445 (26.2)	18 (39.1)	32 (45.7)	11 (23.4)	384 (25.0)
55 and over	1067 (62.9)	22 (47.8)	30 (42.9)	28 (59.6)	987 (64.3)
BMI, mean (Sd)	26.5 (4.1)	27.1 (5.1)	26.7 (4.9)	26.2 (3.3)	26.5 (4.1)
BMI > 30	320 (18.9)	13 (28.3)	18 (25.7)	8 (17.0)	281 (18.3)
Deliveries					
Cesarean Sections	115 (6.8)	4 (8.7)	7 (10.0)	4 (8.5)	100 (6.5)
Vaginal deliveries	1580 (93.1)	39 (84.8)	62 (88.6)	44 (93.6)	1435 (93.5)
Nulliparous or status unknown	102 (6.0)	5 (10.9)	6 (8.6)	2 (4.3)	89 (5.8)
1–2 vaginal deliveries	872 (51.4)	23 (50.0)	35 (50.0)	22 (46.8)	792 (51.6)
3 or more vaginal deliveries	708 (41.7)	16 (34.8)	27 (38.6)	22 (46.8)	643 (41.9)
Preceding operations					
Preceding POP operation	38 (2.2)	1 (2.2)	3 (4.3)	2 (4.3)	32 (2.1)
Any preceding operation	655 (38.6)	24 (52.2)	43 (61.4)	23 (48.9)	565 (36.8)
Hysterectomy-related factors					
Main indication					
Fibroids	85 (5.0)	24 (52.2)	17 (24.3)	3 (6.4)	41 (2.7)
AUB	62 (3.7)	5 (10.9)	10 (14.3)	4 (8.5)	43 (2.8)
Dysmenorrea	11 (0.6)	1 (2.2)	2 (2.9)	2 (4.3)	6 (0.4)
Endometriosis	5 (0.3)	2 (4.3)	0 (0.0)	0 (0.0)	3 (0.2)
Adnexal mass or other	51 (3.0)	12 (26.1)	19 (27.1)	9 (19.1)	11 (0.7)
POP	1486 (87.6)	2 (4.3)	22 (31.4)	29 (61.7)	1433 (93.4)
Uterus size, median in grams (IQR)	103 (94)	296 (280)	171 (106)	136 (84)	94 (73)
Uterus > 400 g	24 (1.4)	12 (26.1)	2 (2.9)	0 (0.0)	10 (0.7)
Operations					
Any concomitant operation	1447 (85.3)	39 (84.8)	62 (88.6)	44 (93.6)	1302 (84.9)
Bilateral salpingectomy	105 (6.2)	22 (47.8)	34 (48.6)	27 (57.4)	22 (1.4)
SUI operation (TOT or TVT)	65 (3.8)	3 (6.5)	3 (4.3)	2 (4.3)	57 (3.7)
Any concomitant POP operation	1363 (80.3)	14 (30.4)	44 (62.3)	36 (76.6)	1269 (82.7)
Anterior repair	1137 (67.1)	5 (10.9)	24 (34.3)	28 (59.6)	1081 (70.5)
Posterior repair	846 (49.9)	12 (26.1)	28 (40.0)	21 (44.7)	785 (51.2)
Transvaginal mesh	31 (1.8)	0 (0.0)	0 (0.0)	0 (0.0)	31 (1.8)
Sacrospinous fixation	37 (2.2)	0 (0.0)	1 (1.4)	1 (2.1)	35 (94.6)
Operator status					
Specialist	1146 (67.5)	35 (76.1)	55 (78.6)	34 (72.3)	1022 (66.6)
Resident	450 (26.5)	9 (19.6)	11 (15.7)	9 (19.1)	421 (27.4)
not available	101 (6.0)				
Complications					
Minor	167 (9.5)	14 (30.4)	9 (12.9)	4 (8.5)	140 (9.0)
Major	36 (2.1)	1 (2.2)	4 (5.7)	0 (0.0)	31 (2.0)
Operating Hospital					
University	521 (30.7)	10 (21.7)	39 (55.7)	10 (21.3)	462 (30.1)
Central	739 (43.5)	21 (45.7)	28 (40.0)	28 (59.6)	662 (43.2)
Local	429 (25.3)	15 (32.6)	3 (4.3)	9 (10.1)	402 (26.2)
Private	8 (0.5)	0 (0.0)	0 (0.0)	0 (0.0)	8 (0.5)

*AUB* Abnormal uterine bleeding, *BMI*, Body mass index, *Std* Standard deviation, *SUI* Stress urinary incontinence, *TOT* Transobturator tape, *TVT* Tension free vaginal tape, *AH* Abdominal hysterectomy, *LH* laparoscopic hysterectomy, *LAVH* Laparoscopic assisted vaginal hysterectomy, *VH* Vaginal hysterectomy, *SAH* Subtotal abdominal hysterectomy

^a^All hysterectomies

^b^including SAH and conversions

**Fig. 1:**
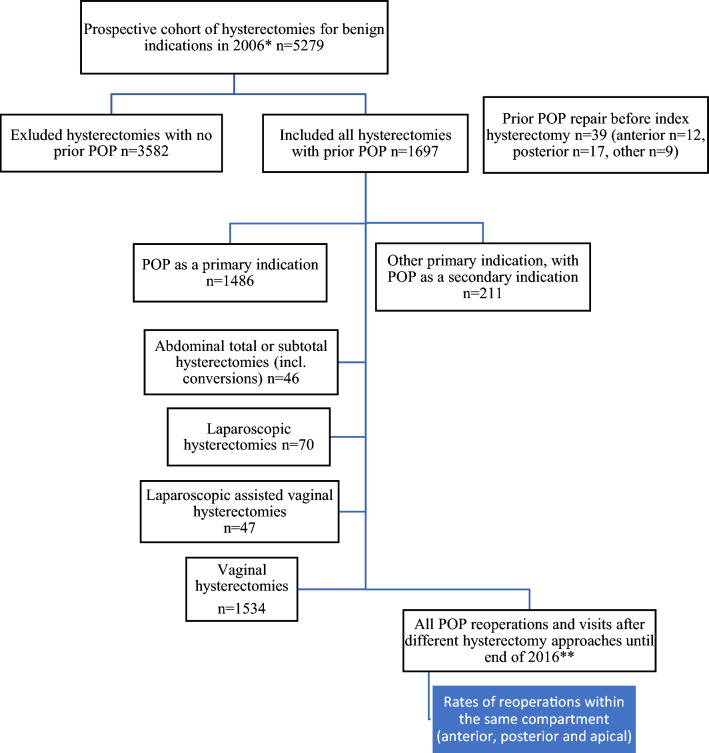
Flow of the sample collection. *POP *Pelvic organ prolapse. *By data collected in the prospective cohort. **By data from the Care register for Health Care

From this dataset we identified 1697 (32%) women who underwent hysterectomy for POP or had some form of POP diagnosis (N81*) or a concomitant operation code for POP (LEF*) within 10 years before or at time of the hysterectomy. Subsequent post-hysterectomy operations and outpatient clinical visits for POP were tracked until the end of 2016. The flow of sample collection is presented in Fig. 1.

We linked the cohort to the Finnish Care Register (HILMO) maintained by the Finnish Institute for Health and Welfare. This register contains information on hospital admission and discharge dates, diagnoses coded according to the International Classification of diseases (ICD) and operation codes based on the Nordic Classification of Surgical Procedures (NSCP) for all clinical in- and outpatient visits in specialized healthcare facilities throughout Finland. From the Care Register, we identified all POP diagnoses (ICD-10 N81*) and/or POP operations (NSCP codes LEF*) from 1996 until the end of 2016. While the Care Register allowed us to identify the affected prolapse compartment, it did not provide information on the stage of prolapse.

We categorized the hysterectomy approaches into abdominal (AH, including both total and subtotal hysterectomies and conversions), laparoscopic (LH), laparoscopic-assisted vaginal (LAVH) and vaginal (VH). The choice of hysterectomy approach was made by the surgeon based on anatomical findings and clinical indications. The surgeon provided the diagnosis, yet not the stage of the prolapse or the method of the vaginal vault suspension.

Data analysis was conducted using IBM SPSS Statistics 28.0. The primary outcome was the rate of post-hysterectomy POP reoperation and/or POP diagnosed at an outpatient clinical visit occurring 60 days or more after the index hysterectomy. We evaluated both the overall reoperation rate and the rate of reoperations within the same compartment. POP reoperations and diagnoses were categorized into three groups based on the affected compartment (anterior, apical, and posterior). We also calculated the number of second and third reoperations divided according to compartments.

To calculate the proportions of first reoperation for each compartment, all POP procedures were included considering that some women underwent surgery in multiple compartments. Type of POP surgery as follows:

1. Posterior vaginal prolapse (N81.6, rectoceles).
2. Anterior vaginal prolapse (N81.1, cystoceles).
3. Vaginal vault prolapse (N99.3; includes enteroceles, N81.5).
4. Multicompartment prolapse (posterior and/or anterior and/or vaginal vault prolapse).

The crude risk for POP (reoperations or visits) was analyzed according to the main indication of the hysterectomy (POP and other indications), and patient or operation-related factors such as the hysterectomy approach, preceding and concomitant operations (prior POP surgery within 10 years before hysterectomy, concomitant anterior and/or posterior repair, sacrospinous fixation (SSF), mesh repair), age, body mass index (BMI), parity, vaginal deliveries, uterus size, some autoimmune disease, chronic obstructive pulmonary disease (COPD)/asthma, intra- and perioperative complications, experience of the surgeon, operator status and hospital type (data not shown).

The variables found to be significant in univariate models (concomitant anterior and posterior repairs, SSF operation, uterus size, previous cesarean section, BMI, operator status) were tested in a multivariate Cox regression model and adjusted with each other, age, hysterectomy approach (with AH as the reference) and vaginal deliveries. For continuous variables, results were expressed as mean and standard deviation (Std) or as median and interquartile range (IQR), with significance determined using the independent t test. For ordinal variable, proportions were calculated using the Chi-squared or Fisher’s exact test as appropriate. When comparing more than two groups, the one-way ANOVA was employed. A significance level of p < 0.05 was used for all analyses.

The study protocol received approval from the Ethical Committee of the Helsinki and Uusimaa Hospital District (Dnro 457/E8/04 on December 16, 2004, and 343/13/03/03/2015 on March 3, 2015). The study was registered in the Clinical Trials (NCT00744172). The Finnish Institute for Health and Welfare of Finland authorized the use of the data from the Care Register (THL/986/5.05.00/2018). The research adhered to the principles outlined in the Declaration of Helsinki and local statutory requirements, with all participants providing written informed consent to participate in the study.

## Results

The mean age of all women was 59.2 years, with the youngest age group being those who underwent LH (Table 1). Among the women who had AH, a significantly lower percentage (84.5%) had experienced vaginal deliveries compared to those who had VH (93.5%) (p = 0.02). Additionally, women who opted for the VH approach had the fewest prior operations compared to other groups (p < 0.01, VH vs. LH). POP served as the most common primary indication for hysterectomy in all cases except for AH, where fibroids were the most common primary indication (p < 0.01). Concomitants prolapse repairs were most frequently performed for the VH approach (p < 0.01, VH vs. AH). The LH approach was most prevalent in university hospitals (p < 0.01, LH vs. others). Among 1697 women, subtotal hysterectomy was performed only on six (0.4%). Of these, one (0,4%, 1/280) required a reoperation for POP, and another (0.3%, 1/359) necessitated a POP-related visit.

**Fig. 2:**
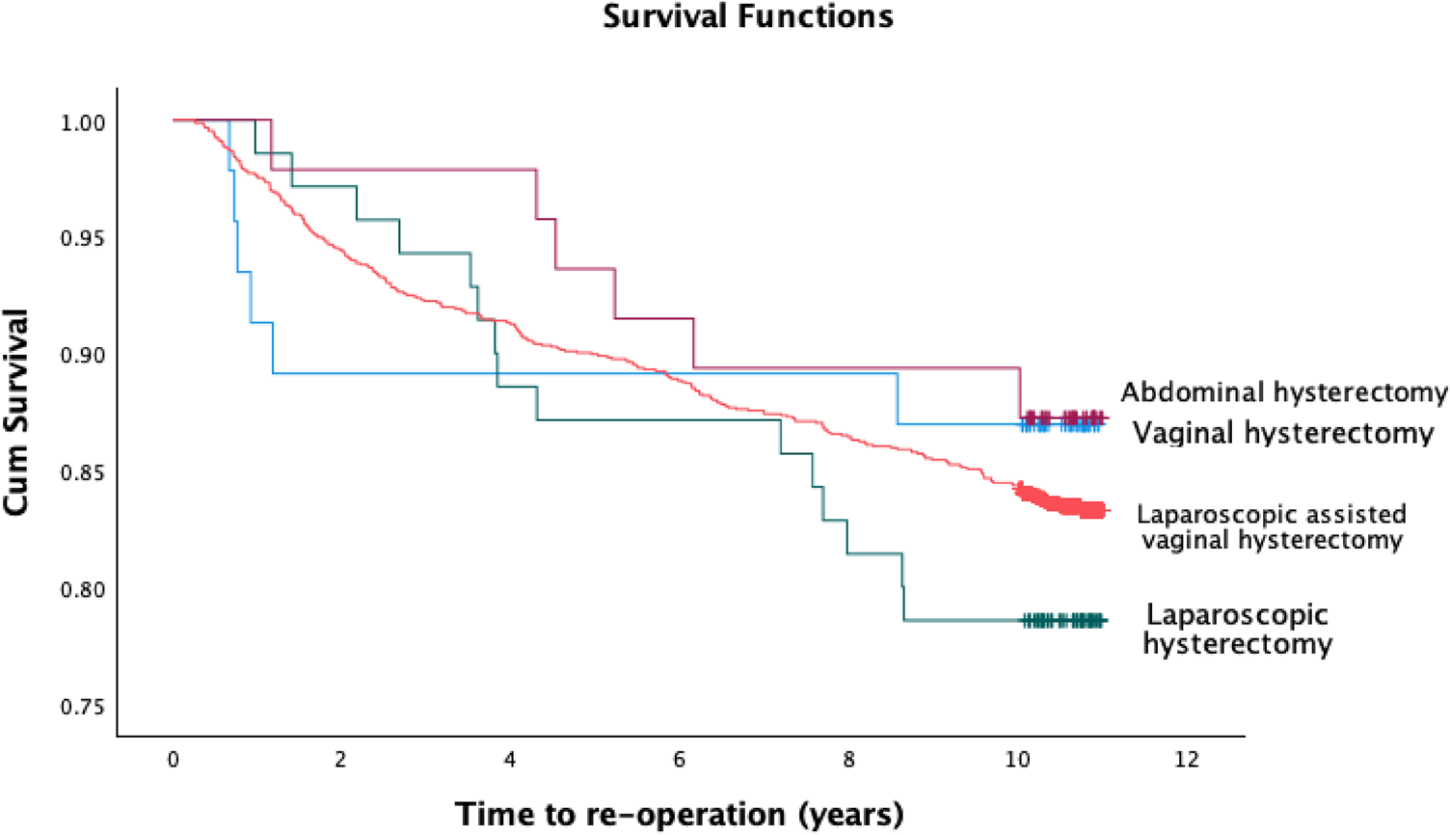
Kaplan–Meier cumulative survival without any POP reoperation after hysterectomy during more than ten years of follow-up time

During the ten-year follow-up time, 280 (16.5%) women had some POP reoperation and 359 women (21.2%) some POP visit (Table 2). The highest number of POP reoperations (n = 15, 21.4%) and visits (n = 17, 24.3%), were among LH approach, yet the difference was not statistically significant (Fig. 2). The most common POP reoperation was the vaginal vault repair (n = 181, 10.7%), followed by the anterior repair (n = 120, 7.1%). A median time to POP repair was 3.7 years (IQR). The most common reason for POP-visits following hysterectomy was cystocele (12%), followed by the vaginal vault prolapse (11%). A median time to any prolapse visit was 3.0 years (IQR). Altogether 23 of 280 women (8.2%) underwent a second reoperation which was only 1.4% of all women (23/1697). These were mostly vaginal vault repairs (9.4%, 17 of 181 primary vault repairs, 1.0%, 17 of 1697 all women). A total of three women (1.1%, 3/280 and of all women 0.2%, 3/1697) underwent a third reoperation.

**Table 2:** Incidence and type of prolapse reoperations and visits after different hysterectomy approaches

	All hyst	AH*	LH	LAVH	VH	
	*n* = 1697	*n* = 46	*n* = 70	*n* = 47	*n* = 1534	*p*
Any POP reoper						
Anterior repair	280 (16.5)	6 (13.0)	15 (21.4)	6 (12.8)	253 (16.5)	*p* = 0.55
Posterior repair	120 (7.1)	3 (6.5)	8 (11.4)	3 (6.4)	106 (6.9)	*p* = 0.55
Vaginal vault repair	76 (4.5)	2 (4.3)	6 (8.6)	1 (2.1)	67 (4.4)	*p* = 0.34
Time to reoperation	181 (10.7)	3 (6.5)	9 (12.9)	3 (6.4)	166 (10.8)	*p* = 0.55
yrs, median (IQR)	3.7 (1.4–6.8)	0.8 (0.7–3.0)	3.8 (2.7–7.8)	4.9 (3.5–7.1)	3.4 (1.5–6.8)	*p* = 0.08*
POP visits						
Cystocele	359 (21.2)	7 (15.2)	17 (24.3)	10 (21.3)	325 (21.2)	*p* = 0.71
Rectocele	204 (12.0)	5 (10.9)	11 (15.7)	5 (10.6)	183 (11.9)	*p* = 0.79
Vag. Vault Prolapse	112 (6.6)	2 (4.3)	8 (11.4)	4 (8.5)	98 (6.4)	*p* = 0.33
Time to visit	186 (11.0)	2 (4.3)	7 (10.0)	5 (10.6)	172 (11.2)	*p* = 0.53
yrs, median (IQR)	3.0 (1.0–6.3)	0.7 (0.5–2.4)	3.6 (1.3–7.1)	3.2 (0.5–6.7)	3.0 (1.1–6.3)	*p* = 0.08*

All hyst, all hysterectomies, AH* abdominal hysterectomy, includes SAH and conversions, *POP* Pelvic organ prolapse, *AH* Abdominal hysterectomy, *LH* Laparoscopic hysterectomy, *LAVH* Laparoscopic assisted vaginal hysterectomy, *VH* Vaginal hysterectomy, *IQR* Interquartile range, *SAH* subtotal abdominal hysterectomy

*significant, * p values from Kruskall-Wallis test for time to prolapse, chi-square and Fischer's exact tests for prolapse type, log-rank test for prolapse visits and repairs and ANOVA test for differences between the groups

When comparing the main pre-hysterectomy POP diagnosis (indication) with the post-hysterectomy one, women with anterior and apical POP required reoperations and visits more frequently (10.4%, 20/192 and 15.4%, 201/1309, respectively. Notably, POP reoperations were often associated with the same compartment that underwent repair concurrent with the hysterectomy (Table 3). Multicompartment reoperations were the most prevalent (11.2%, 153 out of 1363), whereas only 2.7% (23 out of 846) necessitated reoperation for the posterior compartment.

**Table 3:** Numbers of site-specific reoperations

Hysterectomy associated prolapse repair				
POP reoperation n = 280	Anterior *n* = 1137 (%)	Posterior *n* = 846 (%)	Vaginal vault* *n* = 1331 (%)	Multicompartment *n* = 1363 (%)
Anterior *n* = 120	**68 (6.0)**	58 (6.9)	89 (6.7)	89 (6.5)
Posterior *n* = 76	49 (4.3)	**23 (2.7)**	59 (4.4)	58 (4.3)
Vault *n* = 181	114 (10.0)	91 (10.8)	**149 (8.8)**	138 (10.1)
Multicompartment *n* = 202	125 (11.0)	99 (11.7)	165 (12.4)	**153 (11.2)**

*Includes sacrospinous fixations, mesh repairs and hysterectomies with indication as uterus prolapse

Bold value indicates the  percentage of women requiring reoperation within the same compartment.

Among the total cohort, 38 women (2.2%) had previously undergone some POP operation before their hysterectomy (anterior repair 0.7%, 12/1697), posterior repair 1.0%, 17/1697 and other 0.5%, 9/1697), and among this subset, only 4 individuals (10.5%, 4/38 and of all women 0.2%, 4/1697) required a subsequent POP reoperation following the hysterectomy. The incidence of POP related reoperations or visits did not differ significantly between women with a history of pre-hysterectomy POP operation and those who had primary POP repair concomitant with the hysterectomy. Despite the relatively low frequency of POP operations before hysterectomy, 605 women (36%) had received some POP diagnosis during the 10 years before hysterectomy.

In the multivariate analysis, uterine prolapse identified as the primary indication exhibited an elevated risk for any POP reoperation, as well as vaginal vault/anterior reoperation (HR 1.3, CI 95% 1.0–1.6 and HR 1.3, CI 95% 1.0–1.7, respectively) (Table 4). Conversely, a history of cesarean section emerged as a protective factor, reducing the risk for the vaginal vault and anterior prolapse reoperation (HR 0.6, CI 95% 0.3–1.0) and visit (HR 0.5, CI 95% 0.3–1.0), as well as for any prolapse visit (HR 0.6, CI 95% 0.4–1.0). Concomitant anterior repair during hysterectomy reduced risk for the vaginal vault/anterior compartment reoperation and visit (HR 0.7, CI 95% 0.5–0.9 and HR 0.8, CI 95% 0.6–1.0, respectively). Similarly, concomitant posterior repair reduced risk for posterior reoperation and rectocele visit (HR 0.4 CI95% 0.3–0.8 and (HR 0.4, CI 95% 0.3–0–6, respectively), while concomitant sacrospinous fixation correlated with a 2.2-fold risk for vaginal vault/anterior repair (CI 95% 1.2–4.1) and any prolapse repair (CI 95% 1.2–3.9). Uterus weight exceeding 500 g was associated with a substantial 5.0-fold risk for rectocele visits (CI 95% 1.4–18.3). The resident surgeon status presented an elevated risk for any POP reoperations (HR 2.3, CI 95% 1.1–5.0) and visits (HR 2.7, CI 95% 1.3–5.6). The choice of hysterectomy approach did not affect the rate of POP reoperations or visits when adjusting with confounders.

**Table 4:** Multivariate risk analysis for POP reoperations and visits after hysterectomy

Reoperations	Vaginal vault/ anterior repair		Posterior repair		Any prolapse repair	
	HR	95% CI	HR	95% CI	HR	95% CI
Age (yrs)						
< 50	ref		ref		ref	
> 50	1.1	0.8–1.5	1.1	0.6–2.0	1.1	0.8–1.5
BMI						
< 30	ref		ref		ref	
> 30	0.9	0.7–1.3	0.9	0.5–1.6	0.9	0.7–1.3
Uterus size (g)						
< 500	ref		ref		ref	
> 500	1.9	0.6–6.3	4.7	0.9–23.2 (*p* = 0.06)	2.2	0.8–6.7
Cesarean section	0.6	0.3–1.0 (*p* = 0.05)*	1.0	0.4–2.5	0.6	0.4–1.1
Uterine prolapse	1.3	1.0–1.7 (*p* = 0.04)*	0.9	0.6–1.5	1.3	1.0–1.6 (*p* = 0.07)
Vaginal deliveries						
No or unknown	ref		ref		ref	
1–2 deliveries	1.2	0.6–2.0	1.6	0.5–5.1	1.2	0.7–2.3
> 3 deliveries	1.0	0.5–1.7	1.3	0.4–4.4	1.1	0.6–1.9
Operation type *						
AH	ref		ref		ref	
LH	2.3	0.7–7.4	2.6	0.5–14.9	2.0	0.7–6.1
LAVH	1.2	0.3–5.0	0.7	0.1–8.8	1.2	0.4–4.4
VH	1.5	0.5–6.4	1.3	0.3–6.6	1.4	0.5–3.6
Conco AR	0.7	0.5–0.9 (*p* = 0.01)*	0.9	0.6–1.4	0.8	0.6–1.0
Conco PR	1.1	0.8–1.4	0.4	0.3–0.8 (*p* < 0.01)*	0.9	0.7–1.2
Conco SSF	2.2	1.2–4.1 (*p* = 0.01)*	1.2	0.2–4.8	2.2	1.2–3.9 (*p* = 0.01)*
Operator status						
Specialist	1.8	0.8–3.8	1.3	0.4–4.3	2.0	0.9–4.3 (*p* = 0.08)
Resident	1.8	1.0–4.6 *p* = 0.06	1.4	0.4–4.7	2.3	1.1–5.0 (*p* = 0.04)*

POP visits	Vaginal vault/ cystocele visit		Rectocele visit		Any prolapse visit	
Age (yrs)	HR	95% CI	HR	95% CI	HR	95% CI
< 50	ref		ref		ref	
> 50	1.3	1.0–1.7	0.9	0.5–1.3	1.2	0.9–1.6
BMI						
< 30	ref		ref		ref	
> 30	0.9	0.7–1.3	0.6	0.3–1.1	0.9	0.6–1.1
Uterus size (g)						
< 500	ref		ref		ref	
> 500	1.1	0.3–5.0	5.5	1.5–19.8 (*p* = 0.01)*	1.8	0.6–5.2
Cesarean section	0.5	0.3–1.0 (*p* = 0.04)*	0.6	0.3–1.6	0.6	0.4–1.0 (*p* = 0.05)*
Uterine prolapse	1.2	0.9–1.5	0.9	0.6–1.3	1.1	0.9–1.4
Vaginal deliveries						
No or unknown	ref		ref		ref	
1–2 deliveries	1.1	0.7–2.0	1.1	0.5–2.5	1.1	0.7–1.8
> 3 deliveries	1.0	0.5–1.7	0.9	0.4–2.2	0.9	0.6–1.5
Operation type **						
AH	ref		ref		ref	
LH	1.9	0.6–6.1	4.9	0.9–26.7 (*p* = 0.07)	1.8	0.7–4.9
LAVH	1.1	0.3–4.3	3.2	0.5–20.8	1.2	0.4–3.8
VH	1.3	0.5–3.8	2.2	0.4–10.4	1.4	0.6–3.3
Conco AR	0.8	0.6–1.0 (*p* = 0.05)*	0.8	0.6–1.2	0.8	0.7–1.0
Conco PR	0.9	0.7–1.2	0.4	0.3–0.6 (*p* < 0.01)*	0.8	0.7–1.0
Conco SSF	1.5	0.8–2.8	1.0	0.2–3.9	1.7	1.0–3.1 (*p* = 0.07)
Operator status						
Specialist	2.4	1.1–5.1 (*p* = 0.03)*	6.7	0.9–48.3 (*p* = 0.06)	2.4	1.2–4.9 (*p* = 0.02)*
Resident	2.8	1.3–6.1 (*p* < 0.01)*	7.8	1.1–57.6 (*p* = 0.04)*	2.7	1.3–5.6 (*p* < 0.01)*

*AH*, abdominal hysterectomy, *LH*, laparoscopic hysterectomy, *LAVH*, laparoscopic assisted vaginal hysterectomy, *VH* Vaginal hysterectomy *POP* Pelvic organ prolapse, *BMI* Body mass index, *CI* Confidence interval, HR Hazard ratio, ref reference, *Conco AR* Concomitant anterior repair, *Conco PR* Concomitant posterior repair, *Conco SSF* Concomitant sacrospinous fixation * significant, ** Operation type adjusted with BMI, age, previous cesarean section, main indication as uterine prolapse, uterus size, vaginal deliveries, operator status and concomitant operations

## Discussion

During a ten-year follow-up period after hysterectomy, one in six women required a POP reoperation, and one in five women had a POP-related outpatient visit. The latter observation suggests that conservative treatment may have sufficed for some women. These numbers are considerably higher than those identified in our previous study involving women without pre-existing POP, wherein only 1.6% needed POP operation within the subsequent ten years following hysterectomy [17]. It is also noteworthy that only 2.2% of women underwent some POP operation during the ten years before the index hysterectomy 2006, although 36% of these women had some POP diagnosis. This aligns with previous findings reporting the prevalence of symptomatic POP to vary from 2–8% of adult women [1, 4, 18], and asymptomatic POP up to 50% when based upon pelvic examination [1].

Our data is consistent with previous studies reporting reoperation rates ranging from 10–19% [15, 19–23] with varying follow-up time between 5–32 years. Because damage to the pelvic supportive structures and innervation are established risk factors for primary prolapse and POP, it is likely that hysterectomy may exacerbate existing pelvic floor dysfunction [3]. It is notable that the number of women needing reoperation may even increase with longer follow-up time, as the incidence of POP typically peaks among women over 60–70 years [1, 4, 5], while up to 37% of the women in our study were under 54 years. On the other hand, most of recurrences take place within five years after hysterectomy [9, 15, 21, 22] and in our dataset, the median time for reoperation was three years, suggesting that we likely were able to capture most recurrences.

Vaginal vault prolapse repairs were the most common reoperations (10.7% overall), which is in line with prior studies (6.4–9.9%) [21, 22, 24]. This highlights the importance of reconstructing apical support through vaginal vault suspension to reduce the risk of POP recurrence. According to Eilber et al. [25] the absence of apical support doubles the risk of POP recurrence and performing McCall-type culdoplasty or high uterosacral ligament suspension during any type of hysterectomy, may reduce the risk of apical prolapse [26–28]. Although our data lacks information about vaginal vault suspensions, uterosacral ligament suspensions were likely performed in AH, LAVH, and VH during our study period in 2006, in contrast with the technique of LH where vaginal vault closure were performed without suspensions. This difference may, at least in part, explain why the most POP reoperations and visits were in women with LH, even if the difference for other approaches was statistically insignificant.

Considering the advantages associated with vaginal vault suspension, it was surprising to observe an elevated risk for reoperation in women undergoing concurrent sacrospinous fixation. These women probably had an elevated risk of POP recurrence due to advanced prolapse and weak supporting tissue. Moreover, the failure rate of unilateral sacrospinous fixation is high (40–70%) [29, 30]. According to prior studies VH is associated with risk for POP operations later in life [8, 9, 31] but we found no difference between the approaches. This may be due to the differences in cohort sizes and due to patient selection; in this dataset, we only assessed women with pre-existing POP. Furthermore, we were able to separate LAVH from LH in contrast to Lykke et al. [31]. A recent long-term (17 years) follow-up study supports our data showing that not the hysterectomy approach but POP as an indication expose for recurrence [32].

When evaluating the recurrence in same vaginal wall compartment, the apical compartment exhibited the highest reoperation rate (8.8%), followed by the anterior wall at 6.0% and the posterior wall at 2.7%. The finding that only 2.7% of women with rectocele needed reoperation for the posterior wall aligns with the findings of Lavelle et al. [21] (5.8%) but contradicts those of Löwenstein et al. [19] (13%). Although the number of women requiring reoperation for posterior POP is relatively low, it is noteworthy that in women without pre-existing POP, rectocele is the most common POP following hysterectomy [9, 17, 22, 23]. Furthermore, in our dataset, we observed that uterine weight over 500 g suggested a posterior-wall related visit. While the explanation for this association is not entirely clear, it is possible that factors such as heavy straining on the pelvic floor (e.g., pregnancy, delivery, large uterus) may affect pelvic floor supporting structures, potentially contributing to rectocele. The higher incidence of anterior wall and apical recurrences are likely due to damage to common supporting structures such as uterosacral and cardinal ligaments and pericervical ring. This interpretation is supported by our findings and those of previous studies, such as Manodoro S et al. [19] indicating that women undergoing simultaneous repair of both the apical and anterior compartments experience a decreased risk for reoperation. Our data on the recurrence rates of apex and anterior wall largely correspond with previous studies, reporting rates ranging from 7.4% to 10.8% [20, 21].

In assessing the possible risk factors, we found that cesarean section not only protects for a primary [3, 34], but also for a secondary prolapse. This can be attributed to the fact that the women with cesarean deliveries experience less or no vaginal deliveries, thus avoiding the stretching and damage to pelvic floor structures associated with childbirth [35, 36]. Previous studies have suggested that a high level of expertise in surgeons, surgical skills and techniques involving apical fixation and urogynecologists performing all POP procedures explains a reasonable part of their low reoperation rates [15, 37]. In line with this is our finding that the resident status was associated with increased risk of vaginal vault/anterior reoperation (1.8-fold) and any POP reoperation (2.3-fold).

Our study has limitations. We were unable to identify POP in women who were not referred to specialized health care or were not seeking treatment. Because patients with severe pelvic floor symptoms are more prone to seek treatment [38], it is likely that women with mild or moderate symptoms are missing. However, we believe that the operations and visits represent well symptomatic POP, due to good access to public healthcare in Finland. Moreover, we lacked data on the stage of prolapses and it is possible that women with advanced prolapse had higher rate of POP recurrence. VH approach was used in 90% of the hysterectomies, which might reduce the power of the study and explain the lack of difference between the approaches. However, Gabriel et al. [32] assessed the risk of POP after benign or malign AH, VH and LH and concluded that the approach has no effect on the POP risk. Additionally, hysterectomy approach was not randomized, and selection bias caused by surgeons could not be fully adjusted even if controlling several confounders.

Our study’s strengths were the relatively large-sized cohort with prospectively collected information on several potential confounders and modifiers, ability to analyze separate hysterectomy approaches and including only women with existing POP, clear end-point data from reliable national care register and over ten years of follow-up. The data presented in the previous studies is limited only to women with POP surgeries [9, 15, 32], whereas we were also able to assess POP visits which indicated that almost 25 percent of women with POP diagnosis managed with conservative treatment options.

We found that pre-existing POP, not the hysterectomy approach, was associated with POP recurrence (reoperations and visits) in the hysterectomized women and the high rates of both reoperations and visits indicate that hysterectomy may exacerbate this existing pelvic floor dysfunction. Moreover, apical prolapse repair was the most common reoperation, which underscores the need for vaginal vault suspensions during hysterectomy.

## Data Availability

Data supporting this study cannot be made available due to legal reasons. Please contact HUS Women's Clinic, PL140, 00029 Helsinki.

## Abbreviations

POP: Pelvic organ prolapse
AH: Abdominal hysterectomy
LH: Laparoscopic hysterectomy
LAVH: Laparoscopic-assisted vaginal hysterectomy
VH: Vaginal hysterectomy
BMI: Body mass index
SSF: Sacrospinous fixation
